# Ni-B-PTFE Nanocomposite Co-Deposition on the Surface of 2A12 Aluminum Alloy

**DOI:** 10.3390/ma17133294

**Published:** 2024-07-03

**Authors:** Shunqi Mei, Zekui Hu, Jinyu Yang, Jia Chen, Quan Zheng, Burial Lygdenov, Guryev Alexey

**Affiliations:** 1 Hubei Digital Textile Equipment Key Laboratory, Wuhan Textile University, Wuhan 430073, China; sqmei@wtu.edu.cn (S.M.);; 2 The Advanced Textile Technology Innovation Center (Jianhu Laboratory), Shaoxing 312000, China; 3 School of Mechanical & Electrical Engineering, Zhongyuan University of Technology, Zhengzhou 450007, China; 4 Department of Manufacturing Engineering, Polzunov Altai State Technical University, Barnaul 656038, Russia; 5 Department of Manufacturing Engineering, East-Siberian State University of Technology and Management, Ulan-Ude 670013, Russia; 6 Zhejiang Pinnuo Machinery Co., Ltd., Shaoxing 312500, China

**Keywords:** 2A12 aluminum alloy, spinning cup, composite co-deposition, wear resistance, corrosion resistance

## Abstract

The spinning cup, a crucial component of textile equipment, relies heavily on 2A12 aluminum alloy as its primary raw material. Commonly, electroplating and chemical nickel–phosphorus (Ni-P) plating are employed to improve the surface characteristics of the object. Nevertheless, due to the growing expectations for the performance of aluminum alloys, the hardness and wear resistance of Ni-P coatings are no longer sufficient to fulfill industry standards. This study primarily focuses on the synthesis of Ni-B-PTFE nanocomposite chemical plating and its effectiveness when applied to the surface of 2A12 aluminum alloy. We examine the impact of the composition of the plating solution, process parameters, and various other factors on the pace at which the coating is deposited, the hardness of the surface, and other indicators of the coating. The research findings indicate that the composite co-deposited coating achieves its optimal surface morphology when the following conditions are met: a nickel chloride concentration of 30 g/L, an ethylenediamine concentration of 70 mL, a sodium borohydride concentration of 0.6 g/L, a sodium hydroxide concentration of 90 g/L, a lead nitrate concentration of 30 mL, a pH value of 12, a temperature of 90 °C, and a PTFE concentration of 10 mL/L. The coating exhibits consistency, density, a smooth surface, and an absence of noticeable pores or fissures. The composite co-deposited coating exhibits a surface hardness of 1109 HV_0.1_, which significantly surpasses the substrate’s hardness of 232.38 HV_0.1_. The Ni-B-PTFE composite coating exhibits an average friction coefficient of around 0.12. It has a scratch width of 855.18 μm and a wear mass of 0.05 mg. This coating demonstrates superior wear resistance when compared to Ni-B coatings. The Ni-B-PTFE composite coating specimen exhibits a self-corrosion potential of −6.195 V and a corrosion current density of 7.81 × 10^−7^ A/cm^2^, which is the lowest recorded. This enhances its corrosion resistance compared to Ni-B coatings.

## 1. Introduction

Because of the rapid growth of this technology, the global inventory of air-jet spinning equipment has consistently increased since the start of the 21st century. The spinning cup is a key component of air-jet spinning machines. To preserve the longevity of the air-jet spinning machines and fulfill their high-speed and high-production spinning needs, it is imperative to consistently enhance the material performance, manufacturing precision, and surface quality [1].

Aluminum alloy is a primary material used for the spinning cup, which is a basic component of textile equipment. Upon exposure to the atmosphere, a thin protective passivation film, measuring approximately 250 nm in thickness, will develop on the surface. Because of its low hardness, low melting point, poor wear resistance, and negative electrode potential (about −1.67 V), aluminum alloy is not adequately protected by the passivation film on its matrix in highly corrosive and erosive conditions [2,3]. So the research on surface modification technology is crucial for the application of aluminum alloys.

Anodizing, micro-arc oxidation, chemical plating, and electroplating are surface treatment technologies commonly used to improve the surface hardness of aluminum alloys and enhance their resistance to abrasion. However, when compared to chemical nickel plating, both electrochemical oxidation of aluminum and micro-arc oxidation have certain disadvantages. Controlling the thickness of the oxidized layer is challenging in anodic oxidation and maintenance oxidation processes, and the chemical composition of the oxidized layer may not be adequately stable in certain exceptional circumstances [4,5,6]. Electroplated hard chrome coatings have the ability to increase the hardness and wear resistance of aluminum alloys. However, when compared to Ni-B coatings, there is still a need for additional enhancements in terms of hardness and wear resistance. Furthermore, the electroplating procedure for hard chrome is plagued by the problem of inconsistent coating thickness and also results in significant environmental contamination [7]. This technology has the potential to enhance the development of multi-element alloy coatings, including Ni-P-B and Ni-Fe-P, based on fundamental plating layers like Ni-P and Ni-B. It can also create composite surfaces including Ni-P-Al_2_O_3_ and Ni-P-PTFE. These advancements have promising applications in several fields [8].

Electroless plating technology has led to the emergence of two distinct directions in coatings: composite particle coatings and multi-element alloy coatings, branching out from Ni-P and Ni-B coatings [9]. The preparation method of composite particle coatings primarily involves introducing inert micrometer and nanometer particles into the base electroless plating solutions of Ni-P and Ni-B. This results in the deposition of these functional particles along with nickel–phosphorus and nickel–boron alloys, leading to the formation of high-quality coatings with exceptional characteristics such as exceptional hardness, resistance to wear, self-lubrication, and hydrophobicity. Recently, researchers have produced composite particle coatings including Ni-P-PTFE, Ni-P-SiC, and Ni-P-Al_2_O_3_ [10,11,12,13].

Under acidic plating conditions, Li Jianhua et al. [14] deposited Ni-P diamond coatings on aluminum alloys through composite chemical plating. The study involved pretreatment of aluminum alloys, plating solution components, plating process, and discussed process parameters such as pH value, temperature, loading amount, and heat treatment of the plating solution. Wu Jian et al. [15] used a direct-current plasma jet device to deposit diamond films on YG6-cemented carbide with Ni-P-diamond powder as a transition layer. The findings indicated that the bonding of the coating applied to the substrate using plasma jet deposition in the form of a transitional layer was suboptimal. While it was possible to create diamond films, these films had a tendency to separate from the underlying substrate. Wang Yanjie et al. [16] improved the corrosion resistance and surface hardness of 2A12 aluminum alloy by chemical plating Ni-P in a medium-temperature acidic plating solution. After subjecting the coating to heat treatment at a temperature of 400 °C for a duration of 2 h, the study demonstrated that the hardness of the coating could achieve a value of 408 HV.

The aforementioned investigations demonstrate that chemical plating of Ni-P is a surface treatment method with promising development prospects. It has the capability to significantly enhance the surface hardness, corrosion resistance, and wear resistance of alloys. Nevertheless, there remains a lack of adequate research into the impact of plating solution components on the rate of deposition and hardness of the coating. Additionally, the performance of the coating, including surface hardness and resistance to wear, requires enhancement.

Bonin et al. [17] utilized a novel plating solution formula to create a chemical nickel–boron coating for enhancing the corrosion resistance of low carbon steel. The research findings indicate that these coatings exhibit a somewhat superior level of corrosion resistance compared to traditional coatings that include lead stabilizers. Furthermore, in the work of Pancrecious et al. [18], CeO_2_ nanoparticles were employed by the researchers to augment the corrosion resistance of electroless nickel–boron coatings on 365 aluminum alloy. This approach is commonly employed to enhance the corrosion resistance of many other coatings by utilizing nanoparticles.

In addition, researchers utilized alternative neutral nanoparticles to enhance the corrosion resistance of Ni-B coatings. Krishnaveni et al. [19] employed Si_3_N_4_ nanoparticles to enhance the corrosion resistance of nickel–boron coatings on low-carbon steel. Si_3_N_4_ nanoparticles exhibit exceptional hardness, retain their strength even at temperatures reaching 1200 °C, and possess outstanding stability and resistance to oxidation. An investigation was conducted to examine the corrosion resistance properties of Ni-B coatings that were enhanced with Si_3_N_4_ nanoparticles using electrochemical techniques. The findings revealed that the inclusion of these nanoparticles in the coating led to a decrease in the corrosion current density from 8.41 μA/cm^2^ in Ni-B coatings to 4.42 μA/cm^2^ in Ni-B-Si_3_N_4_ coatings. The incorporation of Si_3_N_4_ nanoparticles altered the microstructure of the coating. Nanoparticles exhibit superior corrosion resistance when compared to microparticles. The purpose of enhancing corrosion resistance is to utilize nanoparticles to occupy both the small and large pores in the coating, therefore augmenting the coating’s density.

Furthermore, SiC nanoparticles have been employed to enhance the corrosion resistance of Ni-B coatings [20]. Incorporating SiC nanoparticles into Ni-B-SiC nanocomposite coatings decreases both the corrosion potential and corrosion current density. Incorporating inert nanoparticles decreases the contact surface area between the metal coating and the corrosive media, resulting in a deceleration of the substrate’s corrosion rate. Numerous researchers have utilized rigid nanoparticles like B_4_C and Al_2_O_3_ to enhance the microstructure and corrosion resistance of coatings. This indicates that using these enhancement techniques can effectively improve corrosion resistance. Research conducted by Wan et al. has demonstrated that including PTFE particles into electroless Ni-B plating on low-carbon steel can enhance the corrosion resistance and tribological features of Ni-B electroless coatings. Polytetrafluoroethylene (PTFE) is utilized extensively in several industrial and chemical sectors owing to its distinctive molecular composition and exceptional characteristics. The molecule consists of a chain-like structure where each carbon atom (C) is bonded to a fluorine atom (F), and the fluorine atoms surround the chain. The structure of PTFE imparts a high level of inertness and stability, resulting in robust resistance to numerous chemicals. PTFE exhibits exceptional chemical inertness, rendering it very resistant to chemical reactions with other substances. It demonstrates remarkable thermal stability, remaining functional across the temperature range of −200 °C to +260 °C for extended periods without any performance degradation. Additionally, PTFE possesses excellent dielectric characteristics. The smooth surface and low coefficient of friction of the material result in reduced friction between surfaces, leading to decreased energy loss and wear [21].

The aforementioned investigations demonstrate that electroless Ni-B plating, as a highly promising approach for surface treatment, can significantly improve the hardness and wear resistance of alloys. Presently, the primary emphasis of study is on the production of Ni-B coatings on materials such as mild steel, with only a few studies investigating the chemical Ni-B plating on 2A12 aluminum alloy. This work specifically investigates the Ni-B-PTFE electroless plating process applied to the 2A12 aluminum alloy. Orthogonal studies are undertaken to identify the appropriate basic plating formulation using chemical Ni-B plating. The use of nanoscale PTFE is intended to enhance the coating’s resistance to wear and corrosion, and potentially decrease the friction coefficient of the Ni-B coating. This study is conducted to investigate the composite electroless plating process of nanoscale PTFE. Subsequently, single-factor experiments are performed to identify the most effective composite electroless plating method. This study focuses on analyzing the surface morphology, coating thickness, elemental composition, adhesion, microhardness, corrosion resistance, and wear resistance of the composite electroless plating layer.

The main contribution of this paper is as follows: for 2A12 aluminum alloy, the method of introducing nanoscale PTFE particles for the preparation of Ni-B-PTFE composite coating is proposed, which enhances the wear and corrosion resistance of 2A12 aluminum alloy matrix and Ni-B coating, and reduces the friction coefficient of the composite coating. The differences between our study and other papers are as follows: at present, other researchers have only involved the preparation of Ni-B plating; research on chemical plating of Ni-B-PTFE on 2A12 aluminum alloy is still very rare and highly insufficient; the influence law of different process parameters on the organization and properties of the plating layer is still to be studied in depth; and the hardness, wear, and corrosion resistance of the plating layer are still to be improved.

## 2. Materials and Methods

### 2.1. Materials

**Figure 1:**
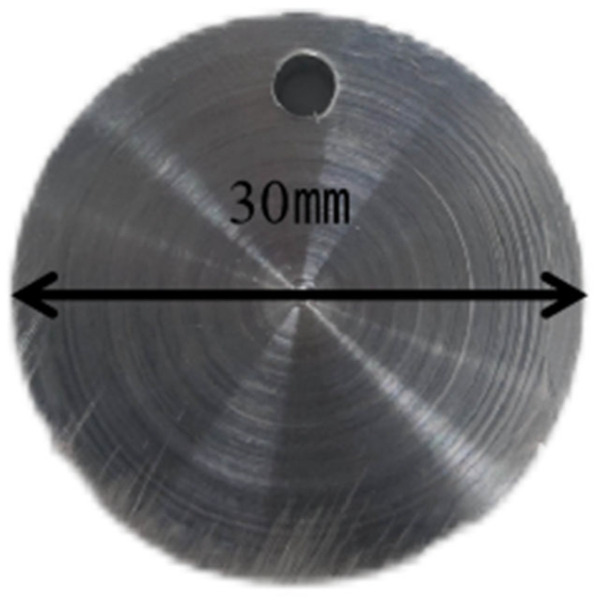
Physical image of standard electroless plating specimens.

Figure 1 displays the typical samples of the experimental materials. The specimens consist of 2A12 aluminum alloy, which serves as the raw material for the revolving cup, a measurement of the content of elements in aluminum alloys by the use of EDX technology. Table 1 displays the composition of the specimen materials. The specimens were fabricated by cutting them into conventional cylindrical shapes with a diameter of 30 mm and a thickness of 10 mm using a DK7740 wire-cutting machine(Jiangsu Taizhou Chuangyuan Machine Tool Co., Taizhou, China). A minuscule circular orifice about 1 mm in diameter was bored in the uppermost part.

### 2.2. Preparation of Ni-B Coatings

The specimens first underwent mechanical leveling using aqueous sandpaper with varying grit sizes (200, 320, 600, 1000, and 2000) to achieve smooth surfaces devoid of obvious scratches. The lye solution was heated to a temperature of 80 °C using a thermostatic water bath. The specimens were then immersed in the solution and left to soak for 10 min. After the soaking period, the specimens were removed from the solution and rinsed with deionized water. A solution of hydrochloric acid with a concentration of 20% was used to immerse the specimens, which were then subjected to activation for a duration of 15 s. The pretreated and activated aluminum alloy specimens were submerged in a zinc solution (composed of NaOH 400 g/L, ZnO 80 g/L, sodium potassium tartrate 8 g/L, FeCl_3_ 1 g/L) for a duration of 40 s. This immersion caused a reaction between the aluminum alloy and the zinc solution, resulting in the formation of a zinc layer that covered the surface of the aluminum alloy. Following a 10-s pickling process using a 50% HNO_3_ solution, the aluminum alloy sample was then submerged in a zinc solution for 15 s following the initial zinc dip. The sample can only be placed in the chemical plating solution for deposition after undergoing a secondary zinc dip. Table 2 provides the process parameters of the Ni-B plating solution.

The steps for preparing the plating solution are as follows:

(1) Sequentially measure the weight of the solid experimental chemicals (nickel chloride, sodium borohydride, sodium hydroxide, lead nitrate) using an electronic scale, following the composition of the Ni-B electroless plating solution. Prior to each weighing, position weighing paper beneath the chemicals, and subsequently, after each chemical is weighed, substitute the weighing paper and cleanse the weighing spoon with anhydrous ethanol.
(2) Transfer the measured substances into separate beakers and then add deionized water until the total volume reaches 50 mL. Next, immerse the beakers in a water bath that is heated by electricity and maintains a consistent temperature. Utilize a glass stirring rod to agitate the mixture until all solid compounds have fully dissolved.
(3) Measure the liquid chemical (ethylene diamine) using a 50 mL graduated cylinder and a glass dropper.
(4) Take a 500 mL beaker, mix the complexing agent (ethylene diamine), stabilizing agent (lead nitrate), and nickel salt (nickel chloride) in sequence, and place it in the electrically heated constant temperature water bath.
(5) Slowly pour the reducing agent (sodium borohydride) into the above 500 mL beaker and continuously stir with a glass rod. Add deionized water until it almost reaches the 500 mL mark.
(6) Insert the magnetic rotor into the 500 mL beaker and position the beaker inside a heat-collecting, constant-temperature magnetic stirrer that has been pre-set to the desired procedure temperature. Adjust the rotational speed to 100 revolutions per minute, and gauge the temperature of the plating solution using a thermometer. When the temperature reaches the desired level, use ammonia water and 10% dilute sulfuric acid to modify the pH value. Once the pH meter reading reaches a steady state at the desired value of the process parameters, promptly introduce the pretreated specimens into the chemical plating solution for deposition.

The experiment utilized an electronic balance with a precision of 0.0001 g to measure the mass of the sample. Additionally, an electronic display thermometer with a precision of 0.1 °C was employed to determine the ambient temperature of the sample. Preliminary tests were conducted prior to the formal experiments, revealing that setting the sample temperature to 90 °C and the rotational speed to 100 rpm resulted in a superior coating quality.

**Figure 2:**
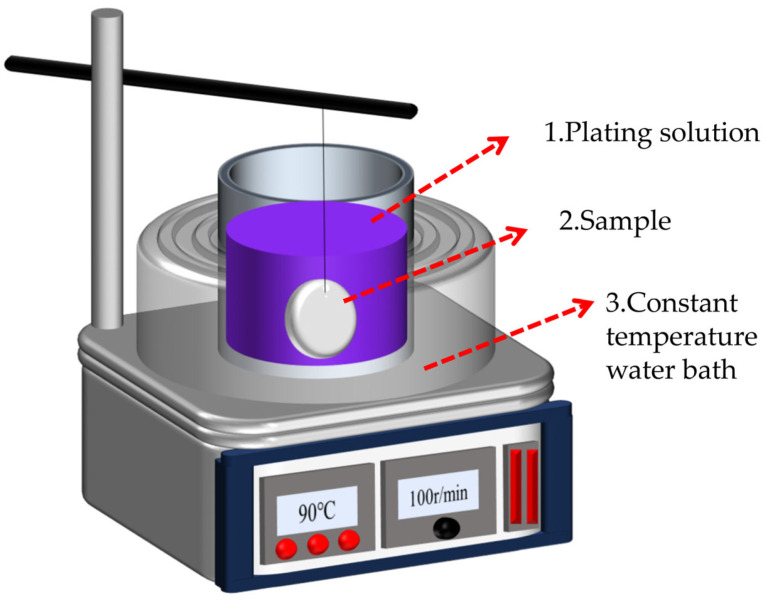
Schematic diagram of Ni-B electroless plating experimental platform.

Figure 2 displays the schematic diagram of the Ni-B electroless plating experimental platform. A heat-collecting, constant-temperature magnetic stirrer provides stable heat energy for the electroless plating. Place the prepared plating solution in a beaker, then immerse the specimens in the water bath, keeping the liquid level of the plating solution 1 cm below the water bath’s level and fully immersing them in the plating solution.

### 2.3. Orthogonal Experiment

The nickel chloride concentration was chosen as 20 g/L, 25 g/L, 30 g/L, and 35 g/L based on the results of a pre-test. It was observed that when the concentration of nickel chloride is below 20 g/L, the concentration of Ni^2+^ is too low, resulting in slower reaction rates and lower quality plating layers. Conversely, when the concentration exceeds 35 g/L, the concentration of Ni^2+^ becomes too high, leading to excessive precipitation of nickel-based compounds at the bottom of the container, which hinders the normal progression of the reaction.

The concentration of ethylenediamine was chosen at 50 mL/L, 60 mL/L, 70 mL/L, and 80 mL/L based on the findings of a preliminary experiment. It was observed that when the concentration of ethylenediamine was below 70 mL/L, the plating rate increased as the concentration of the complexing agent increased. There was a peak in the rate of change in the plating rate and concentration, indicating that the maximum rate occurred when nickel ions were only partially complexed or chelated. Once the concentration of ethylenediamine surpasses 70 mL/L, the maximum value is attained subsequent to a decline in the plating rate of free Ni^2+^.

Sodium borohydride was chosen at concentrations of 0.6 g/L, 0.8 g/L, 1.0 g/L, and 1.2 g/L based on preliminary experiments. It was observed that concentrations below 0.6 g/L did not provide sufficient reductant and Ni^2+^ for the redox reaction. Conversely, concentrations above 1.2 g/L caused instability in the plating solution, negatively impacting the reaction. These studies revealed that a chemical plating period of 1.5 h was found to be the most favorable. This is because the plating solution exhibited instability and a tendency to decompose beyond the 1.5 h mark. The parameters and levels chosen are displayed in Table 3.

### 2.4. Coating Performance Testing

Observation was carried out using a Japanese OLYMPUS-DSX510 metallographic microscope (Yijingtong Optical Technology (Shanghai) Co., Shanghai, China)with a magnification of 693×, 1040×, etc. The thickness of the plated layer was measured and then the chemical deposition rate was determined by metallography. The microhardness of the plated layer was measured using a microhardness tester of model HV1000, with the loading load set at 100 g and the holding time at 10 s, and five points were measured at different positions on the surface of the specimen, and the average value was taken. A JSM-7800F scanning electron microscope was used to further observe the microscopic morphology of the plated section. The tissue structure of the plated layer was analyzed using a Dutch Panaco X-ray diffractometer. The measurement parameters of this experiment were set as follows: Cu target Kα radiation; accelerating voltage 40 kV; working current 40 mA; scanning speed 5°/min; scanning step 0.02°/s; and scanning angle 5–85°. The friction and wear tests were carried out on the specimens using the UMT-3 friction test to test the coefficient of friction and wear resistance of the coatings. The experimental parameters were set as follows: the load force was 10 N, the frequency was 2 Hz, the friction travelling distance was 5 mm, the friction time was 30 mm, and a GCr15 standard steel ball with a diameter of 10 mm was used as the friction vice.

**Figure 3:**
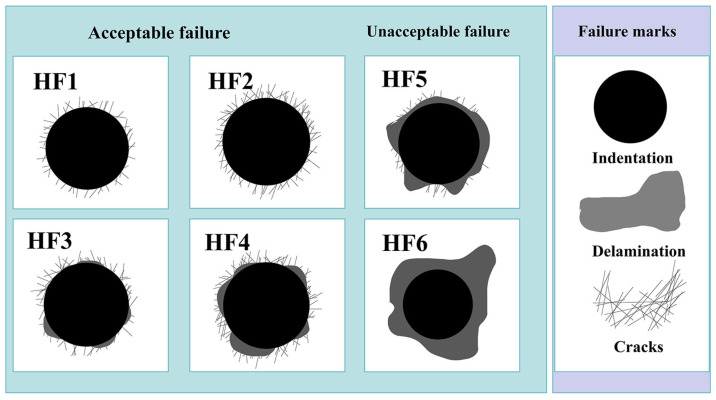
VDI 3198 indentation test principle.

The adherence of the coatings was evaluated by conducting Rockwell hardness (HRC, HR-150A) indentation tests via the VDI 3198 test technique using a 150 kg load. Subsequently, the adhesive strength of the coatings was evaluated in relation to predefined standard values, specifically classified as HF1-HF4 and HF5-HF6, as depicted in Figure 3 [22,23]. The first group suggests acceptable adhesion, while the second group indicates insufficient adhesion.

We evaluated the corrosion resistance of the coating by employing a CH1660E electrochemical workstation. An experimental investigation was conducted at room temperature using a three-electrode system. The working electrode was the specimen, the auxiliary electrode was made of platinum, and the reference electrode was a saturated calomel electrode. The corrosive medium used was a 3.5% NaCl solution. The experiment started with an initial potential of −0.7 V and ended at −0.2 V, with a scanning rate of 5 mV/s.

By analyzing the results of the orthogonal experiment, the optimal chemical plating parameters for Ni-B are determined, providing a basis for exploring the composite electroless plating scheme with nanoscale PTFE.

### 2.5. Preparation of Ni-B-PTFE Coatings

The Ni-B coating was synthesized using an alkaline chemical plating solution containing ethylenediamine as the reducing agent and nickel chloride as the primary salt. The experimental investigation focused on the impact of varying concentrations of nano-PTFE on the morphology and arrangement of the composite plating layer. The concentration of PTFE was the sole factor considered, and the specific process parameters can be found in Table 4.

**Figure 4:**
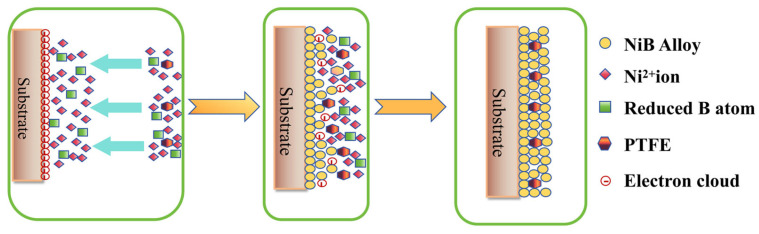
Schematic diagram of PTFE composite co-deposition principle.

Figure 4 depicts the schematic diagram of the PTFE adsorption deposition method. The plating solution contains suspended nanoscale PTFE particles that adhere to the substrate surface as the plating solution flows. Submicron PTFE particles gain a positive charge on the surface of the substrate, become reactive, and then combine with metal ions on the substrate surface to create a composite coating. The proportion of nanoscale particles in the composite coating is influenced by several factors, including the extent of positive charge gained by the particles, their dispersion, the quantity of surface active agent, and the composition of the plating solution [24].

## 3. Results

### 3.1. Stability of the Plating Solution and Macroscopic Appearance Analysis of Specimens

(1) Stability of the Plating Solution and Analysis

**Figure 5:**
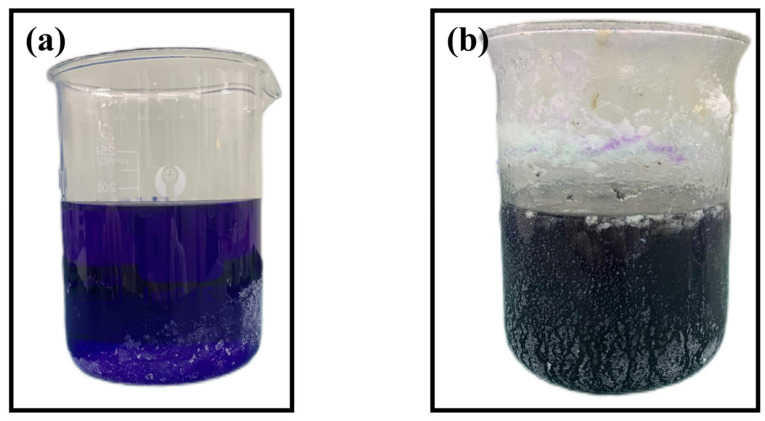
(**a**) Normal electroless nickel plating solution (**b**) Electroless nickel plating solution for decomposition.

Throughout the deposition process, specific constituents within the plating solution may undergo decomposition. This decomposition can be caused by improper concentration configuration of one or more components in the plating solution, or by improper setting of process parameters (such as pH value, temperature, or excessive impurity content) [25]. Such decomposition can lead to uncontrolled reaction rates between Ni^2+^ ions and the reducing agent, resulting in the extensive formation of irreversible nickel foam precipitates, ultimately leading to the failure of the entire chemical plating process. Figure 5a shows the image of a normal Ni-B chemical plating solution, while Figure 5b shows the image of the plating solution when the Ni-B plating solution components decompose.

From the above images a and b, the Ni-B plating solution exhibits a distinct, translucent purple hue. When Ni^2+^ ions are dissolved in water, they appear green. After adding ethylene diamine, the plating solution turns purple. The phenomenon observed when the Ni-B plating solution spontaneously decomposes is the generation of a large number of bubbles, causing the solution to become turbid, with a significant amount of precipitate at the bottom of the beaker. When preparing Ni-B coatings on spinning cups made of 2A12 aluminum alloy, the breakdown of the Ni-B plating solution is represented by T, and F represents no decomposition of the plating solution, then the decomposition status of all 16 sets of orthogonal experiment plating solutions is recorded in Table 5:

(2) Coating Appearance

**Figure 6:**
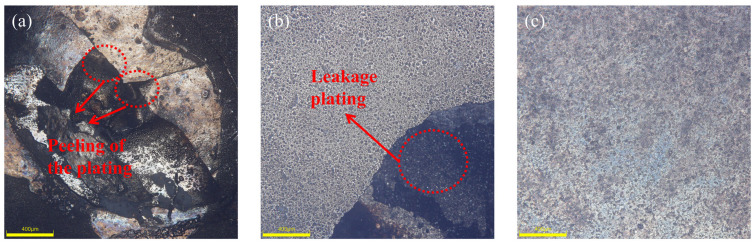
(**a**) Peeling nickel-boron plating, (**b**) Missing nickel-boron plating, (**c**) Normal nickel-boron plating.

By closely examining the visible characteristics of the 2A12 aluminum alloy following the Ni-B chemical plating procedure, one may promptly make an initial assessment of the quality of the resulting Ni-B coating. Indications of subpar coating quality are observed when phenomena such as missing plating, yellow patches, and peeling are present on the surface of the specimen [26]. Upon careful investigation, it was found that the plated layer in group 3 exhibited surface peeling, while group 7 showed leakage plating. However, the appearance of the plated layer in the other 14 experimental groups was superior. Figure 6 shows three samples: (a) represents a sample from group 3 with surface skinning, (b) represents a sample from group 7 with surface leakage plating and poor plating quality, and (c) represents a sample with a bright surface, no leakage plating skinning, and a good macroscopic morphology.

The symbol F is used to denote a specimen that has a glossy surface and shows no signs of missed plating or peeling. On the other hand, the symbol T is used for a specimen that exhibits phenomena such as yellow spots, missing plating, and peeling on its surface. The Table 6 displays the macroscopic appearance records of all 16 sets of orthogonal experiment specimens.

Based on the provided data, it is evident that most of the Ni-B coating samples exhibit a glossy surface without any instances of incomplete plating or peeling, except for specimens 3 and 7, which experienced missed plating and peeling. These results indicate that, regardless of whether the plating solution decomposes, it has little effect on the macroscopic condition of the specimens. As long as appropriate pretreatment processes are followed, generally good macroscopic appearance and better coating quality can be achieved for the specimens.

### 3.2. The Effect of Plating Solution Components on Deposition Rate and Hardness

**Figure 7:**
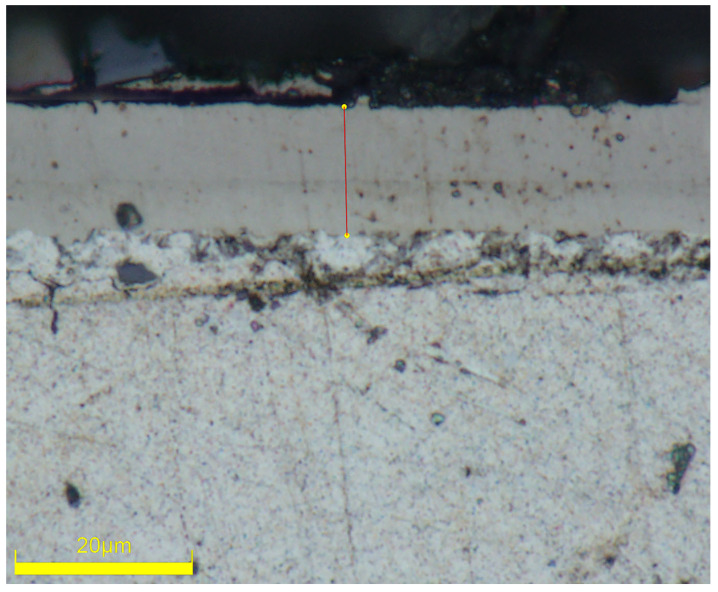
Measurement of Ni-B coating thickness.

(1) The impact of plating solution components on the rate of deposition:

Figure 7 displays the plating measurement results for the 11th set of experimental samples. The thickness of the Ni-B coating was determined by repeatedly measuring it with a metallurgical microscope at a fixed magnification. The average value of each plated specimen was calculated based on five measurements. The plating speed was then quantified as the rate at which the thickness of the plated layer increased over a given unit of time. Table 7 presents the deposition speed records of 16 groups of orthogonal tests, while Table 8 provides a study of the polar deviation using the deposition speed as a measure. The polar deviation, denoted as R, measures the extent of data fluctuation. A large polar deviation indicates a wide range of fluctuation and significant variability between data points. Conversely, a small polar deviation suggests that the values in the dataset are concentrated, resulting in a narrow range of fluctuation. Ki represents the cumulative sum of the levels of different components; ki is the mean of the sum of the factor levels.

**Figure 8:**
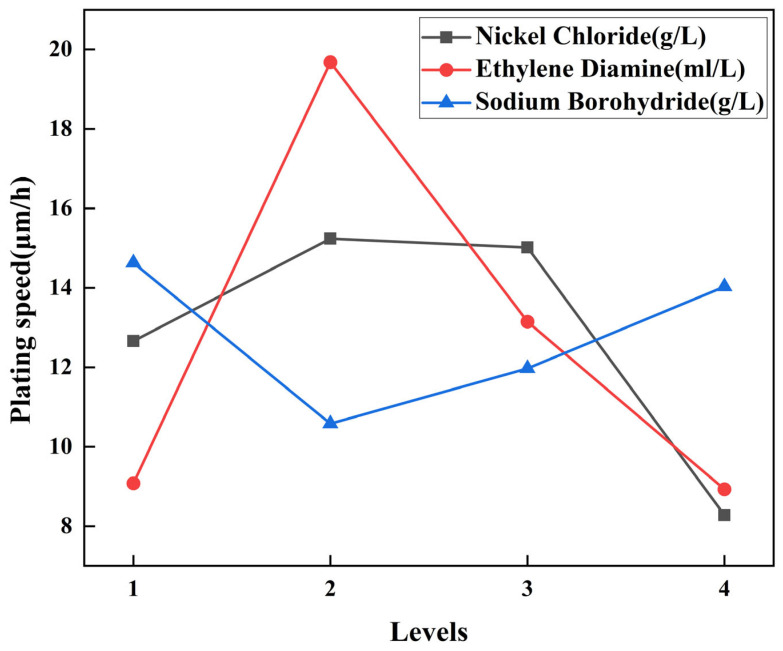
Intuitive effect graph of deposition rate.

The graph in Figure 8 illustrates the inherent impact of each of the three parameters on the rate at which deposition occurs, represented by their respective ki values in the orthogonal experimental design. The deposition rate exhibits an initial increase and subsequent drop as the nickel chloride concentration rises within the range of 20 to 35 g/L, while keeping other parameters constant. The turning point concentration, at which this shift occurs, is 30 g/L. The deposition rate reaches its peak value of 15.24 μm/h when the concentration is 25 g/L. However, when the concentration increases to 35 g/L, the deposition rate reduces to a minimum of 8.28 μm/h. The deposition rate has a non-linear relationship with the ethylene diamine concentration, which ranges from 50 to 80 mL/L. Initially, as the concentration increases, the deposition rate rises, but after reaching a concentration of 60 mL/L, it starts to decline. The deposition rate reaches its peak value of 19.68 μm/h when the concentration is 60 mL/L. However, when the concentration increases to 80 mL/L, the deposition rate reduces to a minimum of 14.03 μm/h. Between sodium borohydride concentrations of 0.6 g/L and 1.2 g/L, while keeping other parameters constant, the rate of deposition initially reduces and then increases as the concentration of sodium borohydride increases. The turning point occurs at a concentration of 0.8 g/L. The deposition rate reaches a peak value of 14.68 μm/h when the concentration is 0.6 g/L, and it decreases to a minimum of 10.58 μm/h when the concentration is 0.8 g/L.

The importance ranking of the three factors on deposition rate is as follows: ethylene diamine > sodium borohydride > nickel chloride. Ethylene diamine has the greatest influence on the deposition rate, while nickel chloride has the smallest influence. The ideal plating solution components and process parameters for achieving the highest deposition rate are as follows: The ideal plating solution components and process parameters for achieving the highest deposition rate are as follows: 90 °C, a pH of 12, 25 g/L nickel chloride, 60 mL/L ethylene diamine, and 0.6 g/L sodium borohydride.

(2) The impact of plating components of solutions on the substrate’s degree of hardness:

In this experiment, the microhardness tester was used to measure the hardness of a specimen. The loading load was set to 100 g and the holding time was 10 s. Five points on the surface of the specimen were measured at different positions, and the average value was calculated [27]. Table 9 displays the experimental findings, whereas Table 10 presents an analysis of the measurement indicator, microhardness, in terms of its range.

**Figure 9:**
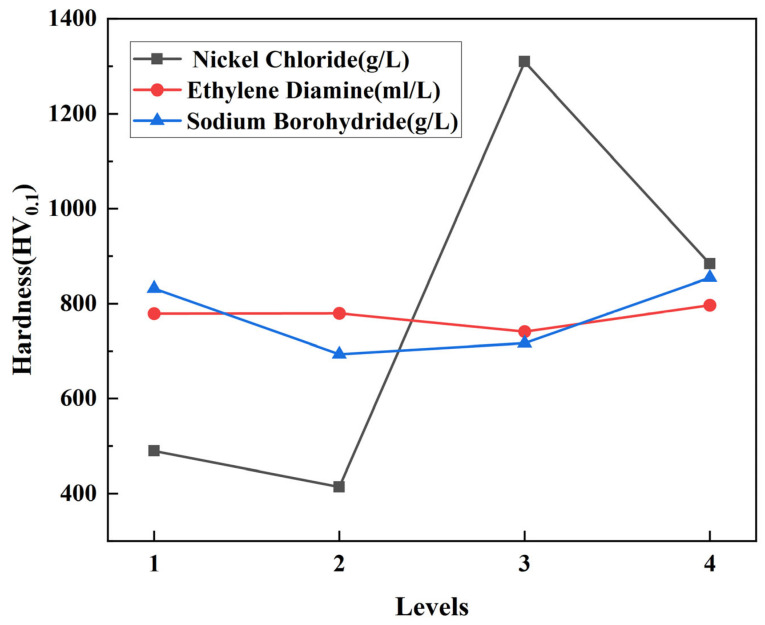
Intuitive effect graph of microhardness.

Figure 9 depicts the direct influence of the three parameters on the deposition rate, indicated by their corresponding ki values in the orthogonal experimental design. From the graph, it can be observed that within the range of 20~35 g/L nickel chloride concentration, with the other factors held constant, as the nickel chloride concentration increases, the hardness initially decreases, then increases, and then decreases again, with a turning point concentration at 25 g/L. At a concentration of 30 g/L, the hardness reaches its maximum value of 1309.54 HV_0.1_, while at a concentration of 20 g/L, the hardness is at its minimum of 414.07 HV_0.1_. Within the range of 50~80 mL/L ethylene diamine concentration, with the other factors held constant, the microhardness remains relatively constant as the ethylene diamine concentration increases, reaching its maximum value of 796.89 HV_0.1_ at a concentration of 80 mL/L, and its minimum value of 741.15 HV_0.1_ at a concentration of 70 mL/L. Within the range of 0.6~1.2 g/L sodium borohydride concentration, with the other factors held constant, the hardness first decreases and then increases, with a turning point concentration at 0.8 g/L. At a concentration of 1.2 g/L, the hardness reaches its maximum of 854.63 HV_0.1_, while at a concentration of 0.8 g/L, the hardness is at its minimum of 693.40 HV_0.1_.

From the analysis of the microhardness range, it can be inferred that the importance ranking of the five factors on microhardness is as follows: nickel chloride > sodium borohydride > ethylene diamine. Nickel chloride has the greatest influence on microhardness, while ethylene diamine has the smallest influence. In terms of hardness, the optimal plating solution components and process parameters are as follows: pH of 12, temperature of 90 °C, 30 g/L nickel chloride, 80 mL/L ethylene diamine, and 1.2 g/L sodium borohydride.

Analyzing the above ranking of primary and secondary factors and the results from the intuitive effect graphs, it can be concluded that among the three factors, nickel chloride has the greatest impact on microhardness. This is because nickel salt is the main source of metal ions in the electroless plating solution, and its concentration directly affects the supply of metal ions. Higher concentrations of nickel salt can provide more nickel ions, which facilitate the formation of dense and uniform coatings, thus increasing the hardness of the coating. Variations in the concentration of nickel salt also impact the chemical reactivity and electrochemical characteristics in the electroplating solution, thus impacting the rate at which deposition occurs. Typically, higher concentrations of nickel salt lead to faster deposition rates, making it easier for metal ions to deposit and form denser coatings, resulting in increased hardness [28].

Changes in the concentration of nickel salt also have an impact on the plating solution’s chemical and physical characteristics, including its pH level and ionic strength, which in turn have an impact on how the nickel ions behave during deposition. An appropriate concentration of nickel salt can provide a favorable deposition environment, conducive to the formation of dense and uniform coatings, thereby enhancing the hardness of the coating.

### 3.3. Adhesion Test of Coating

**Figure 10:**
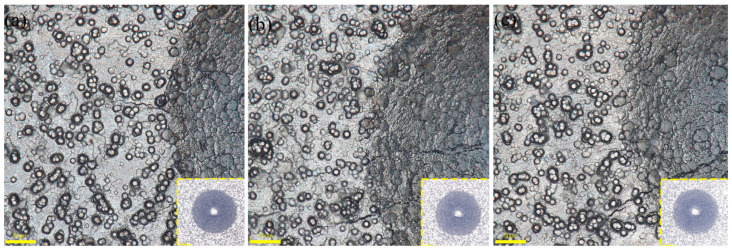
Microscopic appearance of coating adhesion in some samples.

Another crucial indicator of Ni-B coating quality is adhesion. The adherence of the coating is often strong due to the technical properties of chemical plating. In the present investigation, the plated layers were tested for hardness using the Rockwell hardness (HRC, HR-150A) indentation test, following the VDI 3198 test method [22]. After measurement, except for groups 3 and 7, which had poor bonding, the bonding of the remaining 14 experimental coatings was good. As shown in Figure 10, three samples of groups 9, 11, and 12 out of 16 samples are shown. There is no obvious peeling off of the plating on the edge of the scratches of the samples, and the bonding grade is HF1, which indicates that the Ni-B plating has a good bonding force.

### 3.4. Determination of Optimal Preparation Process Parameters for Ni-B Electroless Plating

Based on the summarized influence patterns of each factor on various performance indicators, optimal process parameters for preparing Ni-B coatings on 2A12 aluminum alloy using the rotating cylinder method can be selected [29]. To ensure the stability of the plating solution, experimental sets where solution decomposition occurred (sets 5, 7, and 8) need to be excluded. From the macroscopic appearance of the Ni-B coating samples, sets with surface leakage (set 3) and flaking (set 7) need to be excluded. Regarding deposition rate, faster is not necessarily better, as excessively high deposition rates can lead to uneven metal deposition, resulting in inconsistent coating thickness, affecting product quality and appearance. Excessive deposition rates can also cause excessive hydrogen evolution, forming bubbles during the electroless plating process, making the coating prone to cracking or delamination, affecting its adhesion and stability [30]. High deposition rates also make it more difficult to control the reaction process, making it challenging to predict changes in reaction kinetics, thus complicating the adjustment and control of process parameters [31]. Additionally, excessively high deposition rates may lead to imbalance between metal ions and reducing agents in the solution during the reaction process, affecting the stability and recyclability of the plating solution, and increasing the difficulty of solution management. Therefore, the deposition rate for Ni-B coatings is preferably controlled between 10 μm/h and 15 μm/h [32], excluding sets 1, 9, 11, and 12. In terms of microhardness, higher hardness is preferable. The microhardness of Ni-B coatings in sets 1, 9, 11, and 12 is 401.63 HV_0.1_, 1206.21 HV_0.1_, 1469.47 HV_0.1_, and 1311.27 HV_0.1_, respectively. In the end, as indicated in Table 11, the plating solution constituents and procedure parameters from set 11 of the orthogonal experiment are chosen as the presently ideal ones.

### 3.5. The Microstructure of the Surface and Cross-Section of the Optimal Ni-B Coating Test Sample

**Figure 11:**
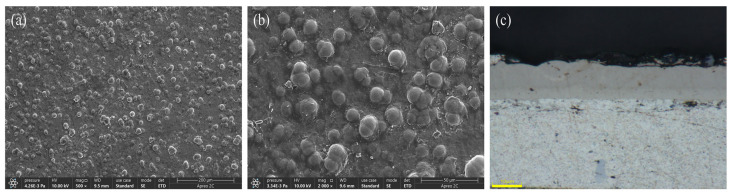
(**a**) Nickel-boron plating at a magnification of 500 times, (**b**) Nickel-boron plating at a magnification of 2000 times, (**c**) Cross-section of the plating under an optical microscope.

Figure 11 displays the plating layers achieved through the optimal Ni-B preparation process chosen in the current orthogonal experiments. Figure 11a, b, and c exhibit the surface micro-morphology under an electron scanning microscope at magnifications of 500× and 2000×, as well as the cross-section of the plating layer under an optical microscope at a magnification of 3000×, respectively. Figure 11a,b depict the surface microstructure of the coating. Following the application of a chemical nickel–boron plating procedure, the surface of the 2A12 aluminum alloy substrate is evenly and entirely coated with a thin layer of nickel–boron alloy. Figure 11c displays the microstructure seen on the cross-section of the sample following the chemical nickel–boron plating treatment. The nickel–boron alloy thin layer has a consistent and smooth structure, firmly bonded to the substrate, suggesting the presence of a robust and compact interface between the nickel–boron alloy thin layer and the substrate. 

### 3.6. Microhardness of the Optimal Ni-B Coating Test Sample

**Figure 12:**
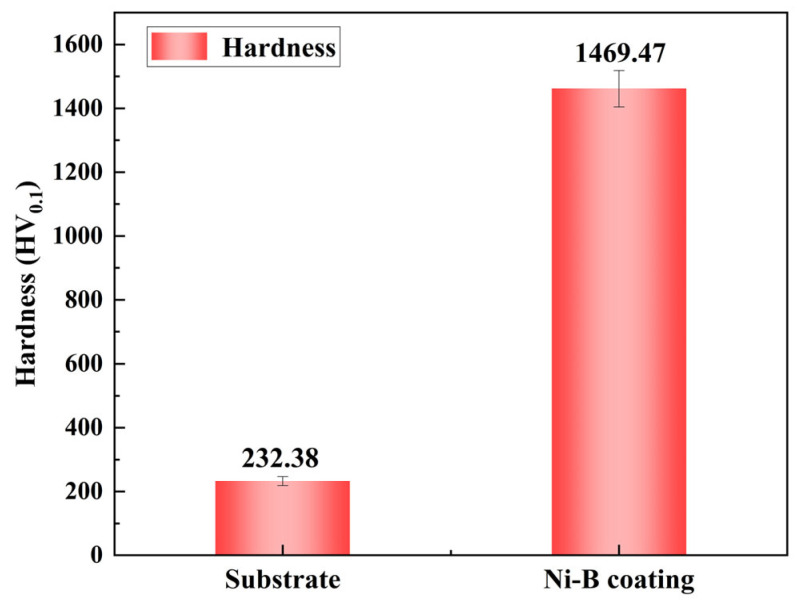
Comparison of microhardness between substrate and the optimal Ni-B coating test sample.

A comparison graph of the substrate’s and coating’s microhardness, derived from the optimal Ni-B preparation method for 2A12 aluminum alloy chosen by the current orthogonal experiment, is shown in Figure 12. The Ni-B coating on the sample in the figure greatly enhances its surface hardness, increasing it from 232.38 HV_0.1_ to 1469.47 HV_0.1_, relative to the substrate.

### 3.7. Energy Spectrum and Microstructure Analysis of the Optimal Ni-B Coating

**Figure 13:**
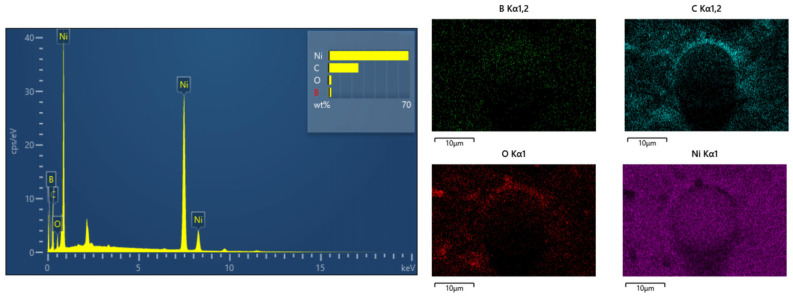
Elemental distribution map of the Ni-B coating.

Using an EDS energy spectrum analyzer to analyze the microstructure and element distribution of the chemically plated Ni-B [33], Figure 13 and Table 12 present the distribution of elements and overall spectra of the Ni-B plating, revealing a B content of 2.54% in the plating. This B content categorizes the plating as a medium boron plating. These visual representations allow us to determine that the coating has 2.54% of boron, which classifies it as a medium boron coating. The coating exhibits a uniform, smooth surface and good brightness. The metal surface becomes very resistant to scratching and wear as a result of the strong hardness of the boron coating; medium boron coatings have good wear resistance and are suitable for applications requiring long-term maintenance of surface smoothness and flatness.

**Figure 14:**
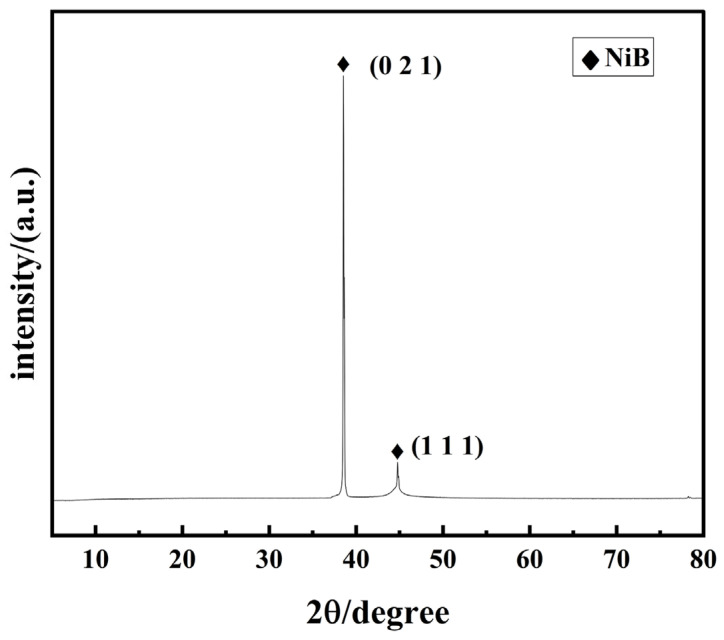
The X-ray diffraction (XRD) spectrum of the most effective nickel–boron (Ni-B) coating applied to the surface of the 2A12 aluminum alloy.

Figure 14 shows the XRD spectrum of the coating obtained from the best Ni-B preparation process for 2A12 aluminum alloy selected by the current orthogonal experiment. The XRD spectrum of the Ni-B coating displays two characteristic peaks, where significant diffraction peaks appeared at 2θ of 38.54° and 44.87°, respectively, which were compared with the standard card (mp-14019) and were found to correspond to the crystal faces of Ni-B at (021) and (111), respectively.

### 3.8. Surface Morphology of Ni-B-PTFE Composite Coating

(1) Appearance of nano-PTFE composite co-deposited coating

**Figure 15:**
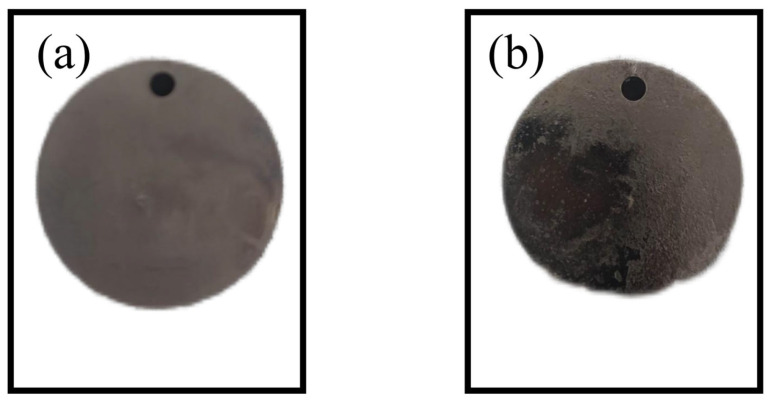
(**a**) nickel-boron coated sample, (**b**) nickel-boron polytetrafluoroethylene coated sample.

The visual examination of the Ni-B-PTFE composite coating surface allowed for the study of its macroscopic morphology, which encompasses characteristics such as color, brightness, and uniformity [34]. Figure 15 depicts the pertinent observation findings, with (a) denoting the Ni-B coating, and (b) denoting the Ni-B-PTFE composite coating. The experimental findings suggest that the composite coating’s surface displays a glossy appearance, with a more compact and even texture, and consistent coverage without any problems of peeling or flaking. Examining the visible structure of the coatings in various settings helps uncover how process parameters affect the coating’s appearance, which is crucial for optimizing the process.

(2) Microstructure

**Figure 16:**
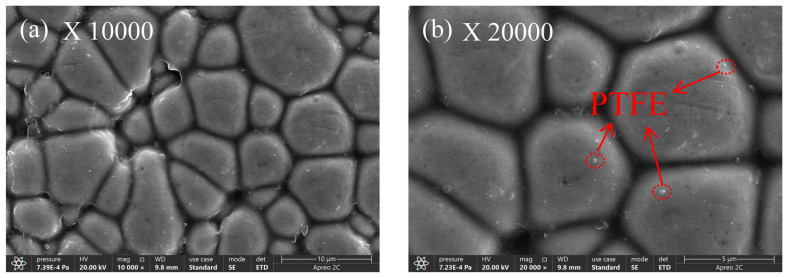
Microstructure of the composite coating with 4 mL/L PTFE concentration.

**Figure 17:**
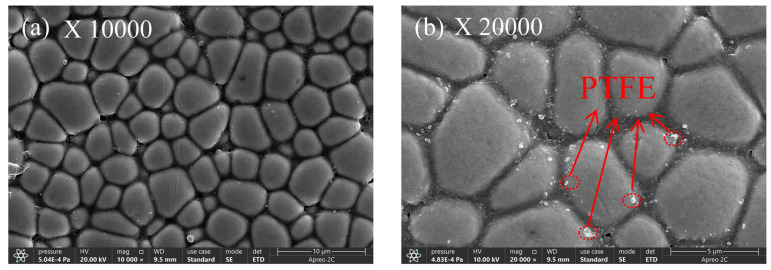
Composite coating microstructure with a 6 mL/L PTFE concentration.

**Figure 18:**
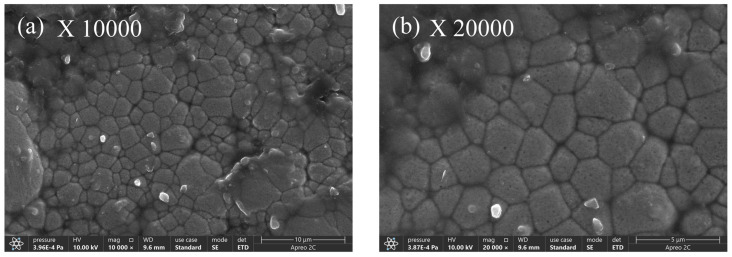
Composite coating microstructure with a 8 mL/L PTFE concentration.

**Figure 19:**
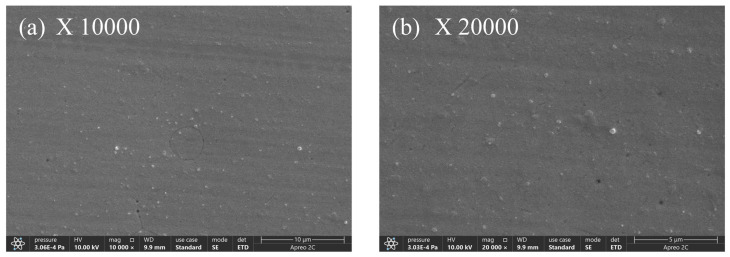
Composite coating microstructure with a 10 mL/L PTFE concentration.

**Figure 20:**
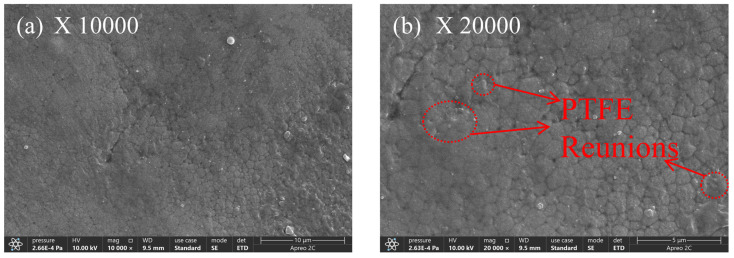
Composite coating microstructure with a 12 mL/L PTFE concentration.

Figure 16, Figure 17, Figure 18, Figure 19 and Figure 20 display the surface microstructure of nano-PTFE composite co-deposited coatings. These coatings were created using various concentrations of PTFE, following the optimal chemical plating conditions for Ni-B. Figure 16 depicts the microstructure of the composite covering at a PTFE concentration of 4 mL/L. Figure 16a,b indicate that the presence of nano-PTFE particles on the coating surface is not substantial, and the coating deposition primarily consists of Ni-B. This phenomenon is caused by the lower amount of PTFE particles present in the coating compared to the amount in the plating solution. This is mainly due to the constant liquid flushing that occurs during the chemical plating process, which prevents the PTFE particles from being deposited on the coating surface [35].

At a PTFE concentration of 6 mL/L, depicted in Figure 17, there is a notable rise in the PTFE content on the coated surface, along with a minor level of aggregation. The reason for this phenomenon is that when the concentration of PTFE increases, the nano-PTFE particles become positively charged on the surface of the substrate and deposit on the substrate surface at the same time as the metal ions. This process initiates the production of a composite coating. Nevertheless, the composite co-deposited coating displays irregular distribution and the presence of visible pores can be noticed in Figure 17b.

When the PTFE concentration is 8 mL/L in Figure 18, as opposed to a PTFE concentration of 6 mL/L, PTFE particles are more noticeable. As the concentration of PTFE increases, there is a notable accumulation of PTFE particles on the surface of the coating. This occurs due to the inadequate dispersion of PTFE particles on the surface of the coating. Nevertheless, Figure 18b shows that more PTFE nanoparticles have been inserted in the Ni-B gaps on the coated surface [36].

Figure 19 shows the physical appearance of the composite co-deposited coating when the concentration of PTFE is 10 mL/L. From Figure 19a,b, it is evident that the PTFE composite coating on the surface of the coating displays homogeneity, high density, lack of pores and cracks, along with a smooth surface and absence of any noticeable flaws. When looking at the microscopic level, the coating quality is best when the concentration of PTFE is 10 mL/L, as compared to concentrations of 4 mL/L, 6 mL/L, and 8 mL/L. The increase in PTFE emulsion concentration and the presence of surfactants result in a higher deposition of PTFE particles on the surface of the composite coating, hence boosting the density of its surface structure [37].

At a PTFE concentration of 12 milliliters per liter, Figure 20 shows the surface morphology of the composite co-deposited coating. There is a discernible aggregation of particles on the coating surface and uneven coverage of the composite coating as compared to a PTFE concentration of 10 mL/L. The findings suggest that as the PTFE concentration increases, the PTFE particles on the coated surface do not fully penetrate the Ni-B coating, resulting in aggregation [38]; the Ni-B coating creates a distinct boundary line and results in a reduced deposition of PTFE particles.

### 3.9. Microhardness of the Ni-B-PTFE Composite Coating

**Figure 21:**
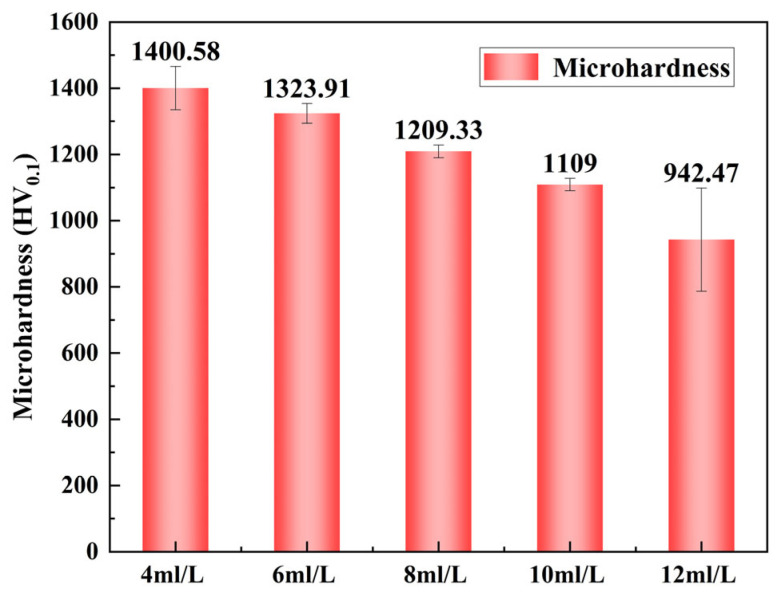
Microhardness of composite coatings with different PTFE concentrations.

Figure 21 indicates that at varying concentrations of PTFE emulsion, the microhardness of the Ni-B-PTFE composite coating exhibits a declining trend. At a PTFE emulsion concentration of 4 mL/L, the coating achieves its highest level of hardness at 1400.58 HV_0.1_. However, when the concentration is increased to 12 mL/L, the hardness of the coating reduces dramatically to 942.47 HV_0.1_. The microhardness of the composite coating is greatly affected by the quantity of PTFE particles deposited. When the amount of PTFE particles being deposited is modest, the load is primarily supported by the Ni-B base coating. This leads to a decrease in plastic deformation and consequently results in a higher surface hardness. The primary factor contributing to the notable reduction in microhardness of the composite coating is the inclusion of PTFE particles, which results in a decline in the strength of the Ni-B coating within the composite. PTFE is a substance that has a low level of hardness. The presence of tiny particles in the composite coating decreases the overall hardness of the coating [39].

### 3.10. Energy Spectrum of Ni-B-PTFE Composite Coating

**Figure 22:**
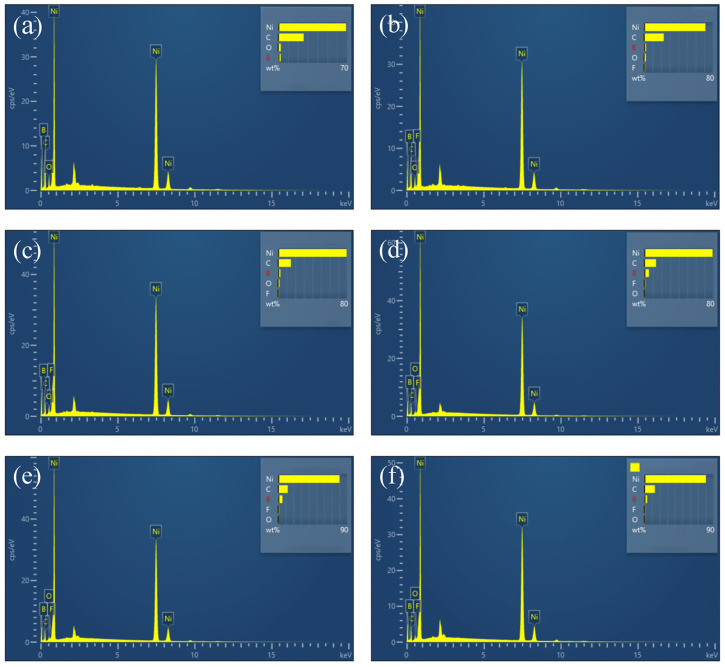
(**a**) Elemental scan of Ni-B coating, (**b**) Elemental scan of composite coating with PTFE emulsion concentration of 4 mL/L. (**c**) Elemental scan of composite coating with PTFE emulsion concentration of 6 mL/L, (**d**) Elemental scan of composite coating with PTFE emulsion concentration of 8 mL/L. (**e**) Elemental scan of composite coating at 10 mL/L PTFE emulsion concentration, (**f**) Elemental scan of composite coating at 12 mL/L PTFE emulsion concentration.

Figure 22 displays the distribution of elements in Ni-B and PTFE composite coatings at various concentrations. Figure 22a shows the elemental scan of Ni-B coating. Figure 22b shows the elemental scan of the composite coating with a PTFE emulsion concentration of 4 mL/L. Figure 22c shows the elemental scan of the composite coating with a PTFE emulsion concentration of 6 mL/L. Figure 22d shows the elemental scan of the composite coating with a PTFE emulsion concentration of 8 mL/L. Figure 22e shows the elemental scan of the composite coating with a PTFE emulsion concentration of 10 mL/L. Figure 22f shows the elemental scan of the composite coating with a PTFE emulsion concentration of 12 mL/L. Based on the findings, it is evident that the largest deposition of PTFE particles on the surface of the composite coating occurs when the concentration of PTFE emulsion reaches 10 mL/L. Additionally, the surface seems to be smooth and thick, with no noticeable imperfections. The EDS scanning reveals that the element distribution of the PTFE coating, at a concentration of 10 mL/L, is homogeneous. The mass percentage of element B is 3.60%, whereas the mass percentage of element F is 1.24%, which is notably greater than the concentrations of PTFE at 4 mL/L, 6 mL/L, 8 mL/L, and 12 mL/L. The results suggest that the distribution of PTFE particles on the coated surface is reasonably uniform, leading to enhanced self-lubricating and anti-friction characteristics of PTFE [40].

### 3.11. Optimal Structure of Ni-B-PTFE Composite Coating

**Figure 23:**
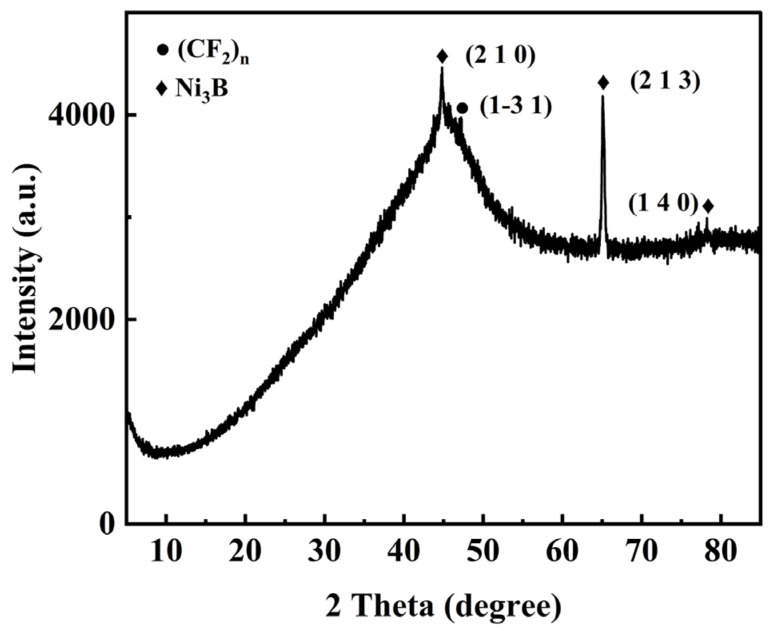
XRD spectrum of the optimal Ni-B-PTFE coating on the surface of 2A12 aluminum alloy.

According to the XRD spectrum in Figure 23, a set of distinctive peaks may be seen in the Ni-B-PTFE composite coating, the figure clearly shows distinct diffraction peaks at 2θ angles of 44.82°, 65.08°, and 78.24°. By comparing these angles with the standard card (mp-2058), it is determined that they correspond to the crystal planes (210), (213), and (140) of Ni3B, respectively. Additionally, by comparing the diffraction peak at 2θ angle of 47.2° with the standard card (mp-559432), it is found to correspond to the crystal faces (CF_2_)_n_ (1−31). According to Bragg’s law, these scattered beams will form diffraction peaks at specific angles. Ni and B, as elements in the crystal, will cause X-ray diffraction due to their atomic arrangement, forming corresponding peaks. However, the peaks of polytetrafluoroethylene (PTFE) shown in the XRD spectrum are not significant because it is an amorphous material and does not have a typical crystal structure [41].

### 3.12. Comparison and Analysis of Friction and Wear Performance

**Figure 24:**
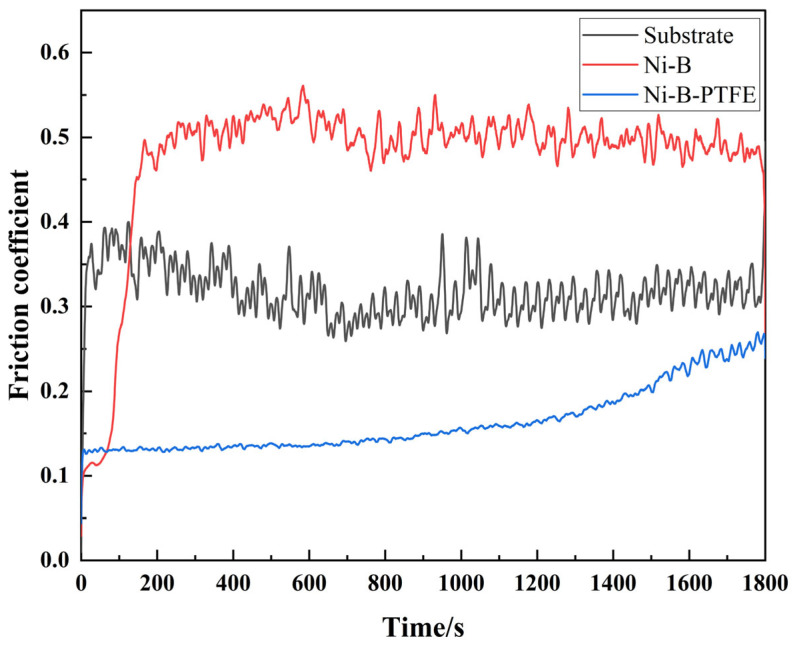
Graph of friction coefficient vs. time relationship.

Figure 24 displays the curve of the friction coefficient of samples undergoing different process treatments over time. The horizontal axis represents time in seconds (s), ranging from 0 to 1800 s, and the vertical axis represents the friction coefficient. The graph shows that the 2A12 aluminum alloy substrate has a comparatively low initial friction coefficient that climbs quickly over time, followed by fluctuating decreases, with an overall increasing trend, ultimately slightly below 0.4. This suggests that there is some variation in the 2A12 aluminum alloy substrate’s friction coefficient. The friction coefficient of the chemically plated Ni-B coating exhibits an initial quick increase, followed by subsequent fluctuations around a value of 0.5. Though more stable overall, the friction coefficient is higher than that of the 2A12 aluminum alloy substrate. This suggests that the Ni-B coating provides relatively good stability in terms of friction performance. The best performance is shown by the chemically plated nano-Ni-B-PTFE composite co-deposited coating, whose friction coefficient rises swiftly at first before falling to about 0.1 and staying there. These findings suggest that the Ni-B-PTFE composite co-deposited coating consistently maintains the lowest and most stable friction coefficient across the full duration of the test. This is because of the special molecular crystal structure of PTFE. When friction has not yet started, the molecular crystal arrangement of PTFE is inconsistent, leading to a relatively high friction coefficient at the beginning of friction. However, as friction continues, the molecular crystal structure of PTFE gradually aligns, leading to a decrease in the dynamic friction coefficient over time, resulting in a final dynamic friction coefficient lower than the initial friction coefficient. This also indirectly reflects the excellent self-lubricating properties of PTFE [42].

**Figure 25:**
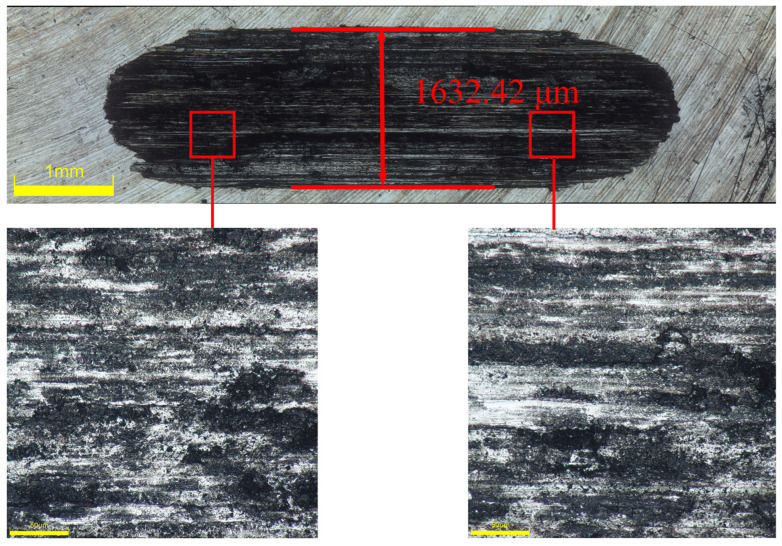
Surface morphology of the wear track on the 2A12 aluminum alloy substrate.

**Figure 26:**
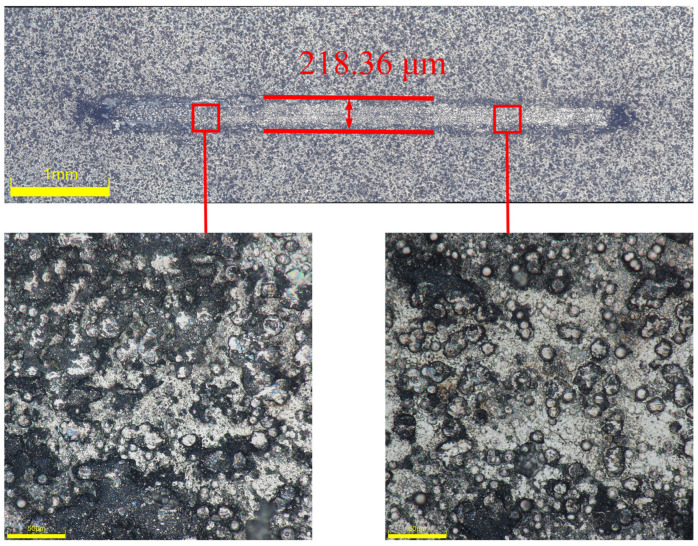
Surface morphology of the wear track on the optimal Ni-B coating of 2A12 aluminum alloy.

**Figure 27:**
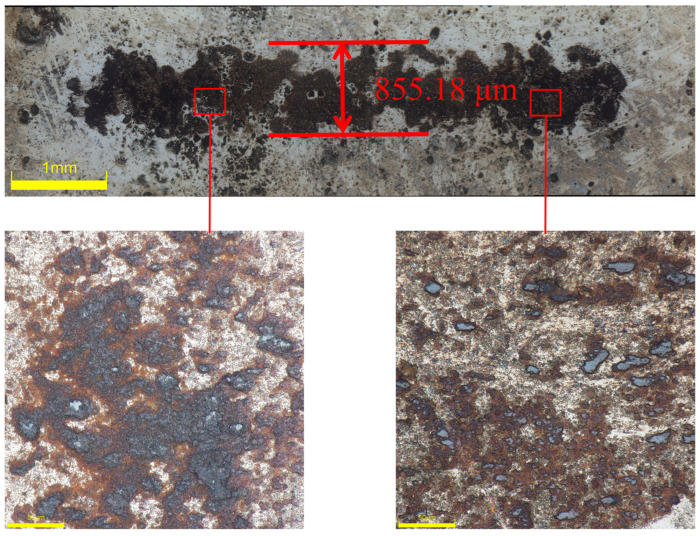
Surface morphology of the wear track on the Ni-B-PTFE coating of 2A12 aluminum alloy.

Figure 25, Figure 26 and Figure 27 show the worn surface morphology of samples after friction for 30 min under a 5 N load after different strengthening processes. Figure 25 depicts the wear track of the 2A12 aluminum alloy substrate under an optical microscope. It can be observed that after 30 min of friction and wear testing, severe wear occurs on the surface of the substrate, displaying distinct wear tracks extending laterally, indicating lateral wear of the material during friction. The width of the wear track is approximately 1632.42 μm, indicating a certain degree of abrasion and cutting on the material surface during friction. In the magnified area, more subtle scratches and plastic deformation can be observed. This is because during friction, high-stress regions are generated at the contact between the mating surface and the substrate, leading to local plastic deformation and abrasion. Additionally, due to the significant difference in hardness between the substrate and the mating surface, abrasion and wear occur on the 2A12 aluminum alloy substrate.

Figure 26 depicts the appearance of the 2A12 aluminum alloy sample following chemical plating with Ni-B treatment. By examining the morphology of the wear track on the surface, it is evident that the width of the surface wear track of the Ni-B plated layer is considerably less than that of the 2A12 aluminum alloy substrate, measuring only 218.36 μm. Simultaneously, there is a notable alteration in the structure of the worn area, resulting in a decrease in depth and a more even surface. The wear resistance of the Ni-B coating is considerably higher than that of the substrate. The wear track has an elongated rod-like shape with rounded ends, which is in line with the expected shape based on theoretical wear track patterns observed in reciprocating friction studies involving steel balls. The upper boundary of the wear track has a white appearance, and there is an absence of a black oxide film at the core, suggesting that the coating has not been completely worn away. Abrasive particles are produced on the Ni-B coating when the coating’s surface is abraded and sliced by the mating surface during friction. The ongoing friction between the mating surface and the coated surface leads to cutting and wear, which worsens the level of wear. The graph reveals a cauliflower-like morphology on the surface of the covering, which appears rough under microscopic view. The results suggest that the friction coefficient of the Ni-B coating is greater than that of the 2A12 aluminum alloy substrate. Additionally, the Ni-B coating exhibits exceptional wear resistance, providing a certain level of protection to the substrate.

Following the application of the Ni-B-PTFE composite coating under ideal processing circumstances, Figure 27 displays the friction and wear morphology of the surface of the 2A12 aluminum alloy substrate. The composite coating method has resulted in a notable improvement in surface-protective qualities, as seen by the thickness and microstructure of the coating. This composite coating demonstrates superior performance in wear compared to earlier treatment methods. The wear track depth is lower than the previous two instances, and it is of moderate depth. The widest width of the wear track is 855.18 μm, indicating the successful protection of the substrate surface by this composite coating. The graph clearly demonstrates that the surface morphology of the nano-PTFE composite co-deposited coating is markedly superior to that of the substrate and chemically plated Ni-B coating. It exhibits the lowest wear depth and shows no noticeable signs of abrasion and cutting. This indicates that the wear resistance and stability achieved through this treatment process are optimal.

**Figure 28:**
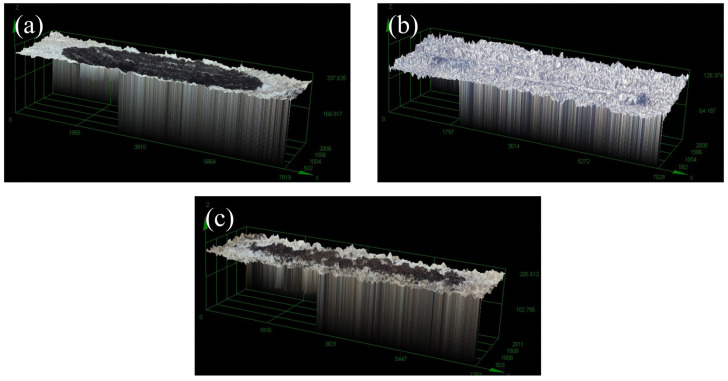
(**a**) 3D abrasion morphology of matrix sample, (**b**) 3D abrasion morphology of nickel-boron coated sample, (**c**) 3D abrasion morphology of nickel-boron PTFE sample.

Figure 28a, b, and c show the 3D wear tracks of the 2A12 aluminum alloy substrate, the best Ni-B coating of the 2A12 aluminum alloy, and the best Ni-B-PTFE coating of the 2A12 aluminum alloy, respectively. The graph clearly demonstrates that the wear depth of the ideal Ni-B-PTFE coating on the 2A12 aluminum alloy substrate is considerably less than that of both the substrate itself and the optimal Ni-B coating. Out of the three samples, the wear track width of the ideal Ni-B coating is the smallest, although its wear mass is not the smallest. The substrate has a wear mass of 0.82 mg, whereas the optimal Ni-B coating has a wear mass of 0.08 mg. The wear mass of the optimal Ni-B-PTFE coating is the smallest, measuring at 0.05 mg.

The results indicate that the sample treated with the chemical plating nano-PTFE composite co-deposition process exhibits the best wear resistance. This is because PTFE has an extremely low friction coefficient, which means it generates almost no friction when in contact with the mating surface. Additionally, PTFE is a chemically inert material that hardly reacts with most chemicals, allowing it to remain stable under various environmental conditions. Furthermore, PTFE possesses excellent flexibility and elasticity, enabling it to adapt to various shapes and stress conditions. This stability under pressure or deformation reduces the likelihood of cracking or wear.

### 3.13. Comparison and Analysis of Electrochemical Corrosion Performance

In order to evaluate the resistance of a spinning rotor made from 2A12 aluminum alloy to corrosion, we conducted a test using chemical plating with Ni-B plating specimen. The plating solution consisted of 30 g/L nickel chloride, 70 mL/L ethylenediamine, 0.6 g/L sodium borohydride, 90 g/L sodium hydroxide, and 30 mg/L lead nitrate. Additionally, we also performed composite co-deposition plating with Ni-B-PTFE, using a base plating and a PTFE concentration of 10 mL/L. The corrosion resistance test was conducted using a corrosive medium consisting of a 3.5% NaCl solution. A corrosion resistance test was performed on the Ni-B plated specimen and the Ni-B-PTFE composite co-deposited specimen. The test used a PTFE concentration of 10 mL/L, 3.5% NaCl as the corrosive solution, an initial potential of −0.7 V, an end potential of −0.2 V, and a scanning speed of 5 mV/s. The corrosion test was conducted at room temperature. The electrochemical characteristics of various samples were assessed by subjecting the test samples’ surface to alternating potentials or alternating currents and measuring the resulting alternating current response on the electrodes [43].

**Figure 29:**
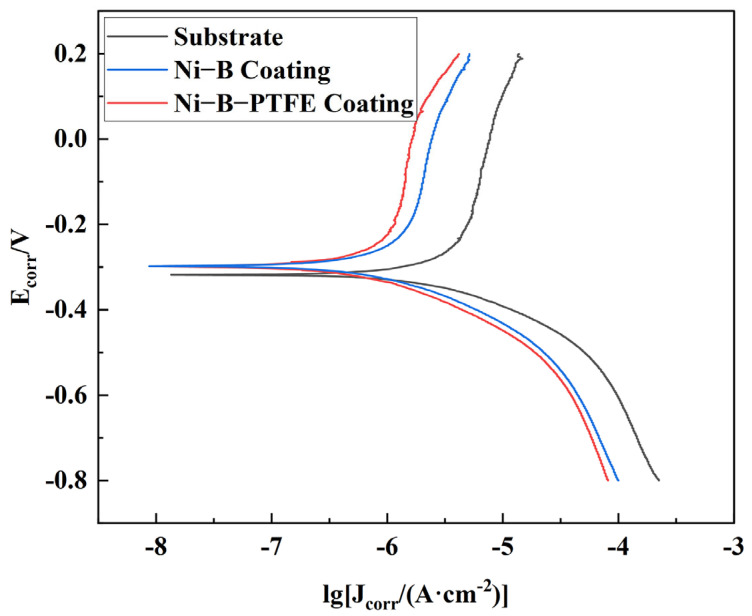
Polarization curves of different samples.

Figure 29 shows the polarization curves of the three samples mentioned above, after the polarization curves were fitted. Table 13 presents the parameters related to electrochemical corrosion. Ecorr and Icorr indicate the corrosion potential and corrosion current, respectively. Cat slp and Ano slp represent the reciprocal of the absolute values of the slopes of the cathode and anode, respectively. Rp denotes the value of linear polarization resistance obtained through the process of fitting.

The corrosion potentials of the three samples, as indicated by the graphs and tables, are −5.607 V, −6.079 V, and −6.195 V. Additionally, the corrosion currents for each sample are 3.376 × 10^−6^ A/cm^2^, 1.087 × 10^−6^ A/cm^2^, and 7.81 × 10^−7^ A/cm^2^, respectively. The polarization resistances for the three samples are 11,783, 36,477, and 51,852 Ω, indicating a consistent pattern of increasing values. Better corrosion resistance performance is shown by a larger polarization resistance, a smaller corrosion current density, and a more negative corrosion potential, which all point to a decreased propensity for corrosion.

The substrate sample has the highest self-corrosion potential, showing its high sensitivity to corrosion. On the other hand, the Ni-B-PTFE coating sample displays the lowest self-corrosion potential, indicating its superior corrosion resistance. The substrate sample exhibits the highest corrosion current density, whereas the Ni-B-PTFE coating sample demonstrates the lowest corrosion current density. This suggests that the Ni-B-PTFE coating sample has the lowest corrosion rate and the highest level of corrosion resistance. The substrate sample exhibits the lowest polarization resistance, whereas the Ni-B-PTFE coating sample demonstrates the highest polarization resistance, suggesting that the Ni-B-PTFE coating sample possesses superior corrosion resistance capabilities.

**Figure 30:**
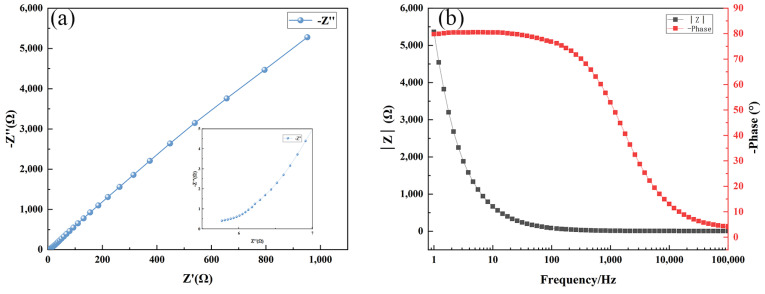
(**a**) Nyquist diagram of 2A12 aluminium alloy matrix sample, (**b**) bode diagram of 2A12 aluminium alloy matrix sample.

**Figure 31:**
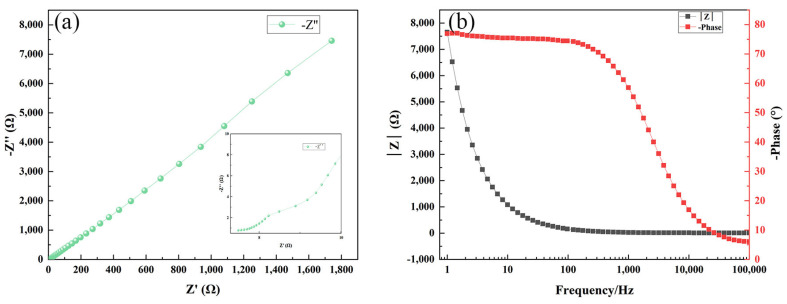
(**a**) Nyquist diagram of Ni-B coated sample, (**b**) bode diagram of Ni-B coated sample.

**Figure 32:**
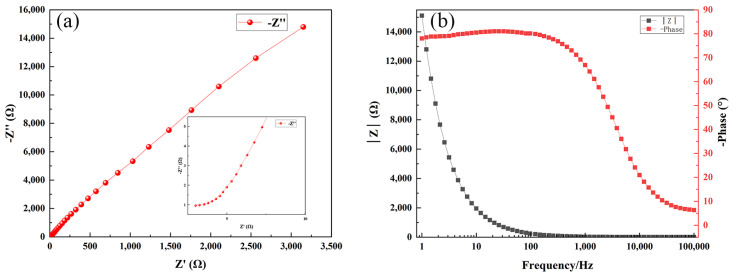
(**a**) Nyquist diagram of Ni-B-PTFE coated sample, (**b**) bode diagram of Ni-B-PTFE coated sample.

The Nyquist and Bode plots of the three samples, which indicate the impedance characteristics of the electrochemical system, are shown in Figure 30, Figure 31 and Figure 32, respectively. The Nyquist plot illustrates the correlation between the imaginary part of impedance (Z″) and the real part (Z′) by graphing them on the complex plane. In this plot, Z′ represents the resistive component. The Bode plot displays the absolute value of impedance, represented as |Z|, and visually represents the correlation between phase angle and changes in frequency. The Nyquist plot facilitates the comparison of the magnitudes of impedance for the three samples. The magnitude of impedance can be assessed by the value of Z′, which indicates the resistive component of the system. The impedance of the Ni-B-PTFE coating sample surpasses 3000 Ω, with the Ni-B coating sample above 1600 Ω. In contrast, the impedance of the substrate sample is the lowest, measuring less than 1000 Ω.

From the Bode plots, it can be observed that the impedance of the substrate sample starts close to 6000 Ω and decreases with increasing frequency, tending to stabilize. The phase angle starts close to 90° and gradually decreases with increasing frequency. The impedance of the Ni-B coating sample starts close to 8000 Ω, but unlike the first two samples, it exhibits an increasing trend at low frequencies before decreasing again. The trend in phase angle change is similar to the first two samples. The impedance of the Ni-B-PTFE coating sample starts close to 14,000 Ω and decreases with increasing frequency. This initial impedance is higher than the first sample, but the trend is similar. The change in phase angle is also similar, starting close to 90° and decreasing with increasing frequency. In summary, the Ni-B-PTFE coating sample exhibits the highest impedance, indicating better corrosion resistance.

## 4. Conclusions

This paper primarily investigates the chemical plating method of nano-Ni-B-PTFE composite co-deposition on the 2A12 aluminum alloy raw material for the spinning cup. It also examines the influence of different process factors on the performance of the coating. Initially, orthogonal tests were performed to investigate the Ni-B basic plating. Subsequently, experiments were undertaken to analyze the effects of various parameters on the coating by co-depositing Ni-B-PTFE composites. Based on the experimental conditions of a nickel chloride concentration of 30 g/L, ethylenediamine concentration of 70 mL/L, sodium borohydride concentration of 0.6 g/L, sodium hydroxide concentration of 90 g/L, lead nitrate concentration of 30 mg/L, pH value of 12, temperature of 90 °C, surfactant content of 30 mg/L, and PTFE emulsion concentration of 10 mL/L, compared to the substrate and the best Ni-B plating, it was found that the Ni-B-PTFE coating exhibited the highest level of wear resistance and corrosion resistance.

The nano-Ni-B-PTFE composite co-deposited coating exhibits superior hardness compared to the Ni-B coating. This allows it to create a robust protective layer on the surface, enhancing the material’s wear resistance. As a result, the wear quality is decreased by almost twice as much. PTFE is added to decrease the coefficient of friction, enhancing the surface’s wear resistance. The coefficient of friction remains at approximately 0.1, as anticipated. Additionally, the inclusion of PTFE allows the composite coatings to demonstrate favorable lubrication properties when subjected to frictional stress, resulting in reduced mechanical wear. The nano-Ni-B-PTFE composite co-deposited coatings exhibit exceptional corrosion resistance, successfully withstanding the erosion caused by different chemical substances and extending the lifespan of the materials.

Research has demonstrated that nano-Ni-B-PTFE composite co-deposited coatings possess the capability to enhance the resistance of materials against abrasion and corrosion. These coatings can be extensively employed in the surface treatment of essential components of textile equipment to enhance their durability and performance.

## Figures and Tables

**Table 1:** Chemical composition of 2A12 aluminum alloy (wt%).

Fe	Si	Mn	Ni	Cu	Ti	Zn	Mg	Al
≤0.5	≤0.5	0.3~0.9	≤0.1	3.8~4.9	≤0.15	≤0.3	1.2~1.8	Bal.

**Table 2:** Composition or process parameters of Ni-B electroless plating solution.

Composition or Process Parameters	Concentration or Parameters
Nickel chloride (NiCl_2_)	20–35 g/L
Ethylene diamine (C_2_H_8_N_2_)	40–80 mL/L
Sodium borohydride (NaBH_4_)	0.6–1.2 g/L
Sodium hydroxide (NaOH)	90 g/L
Lead nitrate (Pb(NO_3_)_2_)	30 mg/L
PH	≥12
Temperature	90 °C

**Table 3:** Factors and levels of Ni-B electroless plating orthogonal experiment.

	Factors	A NiCl_2_ (g/L)	B C_2_H_8_N_2_ (mL/L)	C NaBH_4_ (g/L)
Levels				
1		20	50	0.6
2		25	60	0.8
3		30	70	1.0
4		35	80	1.2

**Table 4:** Ni-B-PTFE composite plating process parameters.

Surface Active Agent Content (mg/L)	PTFE Concentration (mL/L)	Temperature (°C)	pH	Time (min)
30	4	90	12	90
30	6	90	12	90
30	8	90	12	90
30	10	90	12	90
30	12	90	12	90

**Table 5:** Decomposition results of Ni-B electroless plating orthogonal experiment.

Select	1	2	3	4	5	7	8	9	10	11	12	13	14	15	16
Results	F	F	F	F	T	T	T	F	F	F	F	F	F	F	F

**Table 6:** Record of macroscopic appearance in Ni-B electroless plating orthogonal experiment.

Select	1	2	3	4	5	6	7	8	9	10	11	12	13	14	15	16
Results	F	F	T	F	F	F	T	F	F	F	F	F	F	F	F	F

**Table 7:** Results of deposition rate in Ni-B electroless plating orthogonal experiment.

Select	1	2	3	4	5	6	7	8
Results (μm/h)	10.97	18.89	13.68	7.11	3.76	27.71	19.07	10.43
Select	9	10	11	12	13	14	15	16
Results (μm/h)	15.03	23.39	11.67	10.00	6.54	8.72	9.68	8.16

**Table 8:** Analysis results of deposition rate range in Ni-B electroless plating orthogonal experiment.

Code		A	B	C
	Factors	Nickel Chloride (g/L)	Ethylene Diamine (mL/L)	Sodium Borohydride (g/L)
Index				
k_1_		12.66	9.08	14.63
k_2_		15.24	19.68	10.58
k_3_		15.02	13.15	11.97
k_4_		8.28	8.93	14.03
RangeR		6.96	10.60	7.12
Magnitude of Influencing Factors		BCA		
Optimal Combination		A_2_B_2_C_1_		

**Table 9:** Results of deposition rate in Ni-B electroless plating orthogonal experiment.

Select	1	2	3	4	5	6	7	8
Results (HV_0.1_)	402 ± 16	439 ± 24	465 ± 17	653 ± 15	360 ± 40	581 ± 14	367 ± 41	348 ± 19
Select	9	10	11	12	13	14	15	16
Results (HV_0.1_)	1206 ± 25	1251 ± 52	1469 ± 38	1311 ± 24	1148 ± 62	848 ± 31	664 ± 21	875 ± 13

**Table 10:** Analysis results of microhardness range in Ni-B electroless plating orthogonal experiment.

Code		A	B	C
	Factors	Nickel Chloride (g/L)	Ethylene Diamine (mL/L)	Sodium Borohydride (g/L)
Index				
k_1_		489.42	778.91	831.94
k_2_		414.07	779.72	693.40
k_3_		1309.54	741.15	716.70
k_4_		883.64	796.89	854.63
RangeR		895.47	55.74	161.23
Magnitude of Influencing Factors		ACB		
Optimal Combination		A_3_B_4_C_4_		

**Table 11:** Optimal Ni-B electroless plating process parameters for 2A12 aluminum alloy.

Composition or Process Parameters	Concentration or Parameters
Nickel chloride (NiCl_2_)	30 g/L
Ethylene diamine (C_2_H_8_N_2_)	70 mL/L
Sodium borohydride (NaBH_4_)	0.6 g/L
Sodium hydroxide (NaOH)	90 g/L
Lead nitrate (Pb(NO_3_)_2_)	30 mg/L
pH	12
Temperature	90 °C

**Table 12:** Total spectrum map of element distribution of Ni-B coating.

Total Spectrum Map of Element Distribution		
Element	Wt%	At%
B	2.54	6.30
C	25.90	57.86
O	2.57	4.31
Ni	68.99	31.53
Total amount	100.00	100.00

**Table 13:** Fitting results of polarization curves for three samples.

Sample	−E_corr_ (V)	R_p_ (Ω)	I_corr_ (A/cm^2^)	Cat Slp (1/V)	Ano Slp (1/V)
2A12 aluminum alloy substrate	5.607	11,783	3.376 × 10^−6^	8.487	2.444
Ni-B coating	6.079	36,477	1.087 × 10^−6^	8.326	2.641
Ni-B-PTFE coating	6.195	51,852	7.81 × 10^−7^	8.556	2.181

## Data Availability

The datasets used and/or analysed during the current study available from the corresponding author on reasonable request.
